# A comprehensive dataset for home appliance control using ERP-based BCIs with the application of inter-subject transfer learning

**DOI:** 10.3389/fnhum.2024.1320457

**Published:** 2024-02-01

**Authors:** Jongmin Lee, Minju Kim, Dojin Heo, Jongsu Kim, Min-Ki Kim, Taejun Lee, Jongwoo Park, HyunYoung Kim, Minho Hwang, Laehyun Kim, Sung-Phil Kim

**Affiliations:** ^1^Department of Biomedical Engineering, Ulsan National Institute of Science and Technology, Ulsan, Republic of Korea; ^2^The Institute of Healthcare Convergence, College of Medicine, Catholic Kwandong University, Gangneung-si, Republic of Korea; ^3^Center for Bionics, Korea Institute of Science and Technology, Seoul, Republic of Korea

**Keywords:** ERP-based BCI, EEG, transfer learning, BCI dataset, home appliance

## Abstract

Brain-computer interfaces (BCIs) have a potential to revolutionize human-computer interaction by enabling direct links between the brain and computer systems. Recent studies are increasingly focusing on practical applications of BCIs—e.g., home appliance control just by thoughts. One of the non-invasive BCIs using electroencephalography (EEG) capitalizes on event-related potentials (ERPs) in response to target stimuli and have shown promise in controlling home appliance. In this paper, we present a comprehensive dataset of online ERP-based BCIs for controlling various home appliances in diverse stimulus presentation environments. We collected online BCI data from a total of 84 subjects among whom 60 subjects controlled three types of appliances (TV: 30, door lock: 15, and electric light: 15) with 4 functions per appliance, 14 subjects controlled a Bluetooth speaker with 6 functions via an LCD monitor, and 10 subjects controlled air conditioner with 4 functions via augmented reality (AR). Using the dataset, we aimed to address the issue of inter-subject variability in ERPs by employing the transfer learning in two different approaches. The first approach, “within-paradigm transfer learning,” aimed to generalize the model within the same paradigm of stimulus presentation. The second approach, “cross-paradigm transfer learning,” involved extending the model from a 4-class LCD environment to different paradigms. The results demonstrated that transfer learning can effectively enhance the generalizability of BCIs based on ERP across different subjects and environments.

## 1 Introduction

Brain-Computer Interfaces (BCIs) have become a promising technology that establishes a direct communication channel between the human brain and external computational devices (Gao et al., 2021). BCIs have gained attention for their diverse applications across multiple disciplines. Initially developed for medical and rehabilitative purposes, BCIs have expanded to practical areas such as virtual reality, gaming, and robotics (Ahn et al., 2014). One of the primary neural signals used for BCIs is electroencephalography (EEG). BCIs using EEG can be divided into active, reactive and passive BCIs according to the paradigm to generate desired EEG patterns (Amiri et al., 2013). Among them, most reactive BCIs have relied on two major EEG features, event-related potentials (ERPs) and steady-state visually evoked potentials (SSVEPs) (Fazel-Rezai et al., 2012). Specifically, ERP-based BCIs transcend sensory modalities and require less training for the user compared to other methods, making them suitable for real-time interaction with various computer systems (Krol et al., 2018).

The speller is one of the most representative examples of ERP-based BCIs. Over the past 20 years, the BCI speller has served as a communication tool for individuals afflicted with a range of neuromuscular disorders, including ALS, brainstem stroke, brain or spinal cord injury, cerebral palsy, muscular dystrophies, multiple sclerosis, and other patients (Sosa et al., 2011). Moreover, ERP-based BCIs have demonstrated their utility in various domains beyond medical and rehabilitative applications—e.g., game control and lie detection (Marshall et al., 2013; Anwar et al., 2019; Mane et al., 2020). Additionally, they are increasingly being integrated into everyday technologies, providing a hands-free and intuitive means of interacting with computer systems. One emerging area of interest is home automation, where ERP-based BCIs can be employed to control a wide range of household appliances (Bentabet and Berrached, 2016). In addition to the commonly explored applications such as televisions, lighting systems, there exists potential for regulating thermostats, window blinds, and even robotic vacuum cleaners (Kim et al., 2019). For example, a user could effortlessly adjust the room temperature or open the window by simply focusing on specific visual cues presented on a screen. This application not only enhances user convenience but also holds promise for individuals with limited mobility, granting them increased independence in interacting with their home environment.

Despite advancements, ERP-based BCIs face challenges that hinder their widespread adoption and reliability. One of the most pressing issues is inter-subject variability, which refers to variations in ERP responses across different individuals (Pérez-Velasco et al., 2022). Inter-subject variability can be attributed to factors such as age, cognitive abilities, emotional states, and the quality of EEG equipment used (Li et al., 2020). Additionally, ERP responses are sensitive to internal and external conditions, such as the user’s level of focus or environmental noise. Inter-subject variability affects the generalizability and practical utility of ERP-based BCIs, often leading to reduced performance when a BCI model built on pre-existing data is applied to a new user. The complexity of these factors necessitates research into methodologies that can account for inter-subject variability (Maswanganyi et al., 2022). While some progress has been made, such as the development of new signal processing techniques to alleviate inter-subject variability, further development is required (Dolzhikova et al., 2022).

Several studies have attempted to address inter-subject variability in ERP-based BCIs. To address inter-subject variability, the predominant approach is transfer learning, which encompasses a spectrum of methodologies. Among these, Riemannian geometry stands out as a promising mathematical framework for enhancing BCI performance across subjects. It has been utilized for BCI decoding, feature representation, classifier design, calibration time reduction, and specifically, transfer learning, demonstrating its versatility (Congedo et al., 2017). Additional methods include the use of domain adaptation techniques that adjust classifiers to handle new subject data (Jayaram et al., 2016), and the application of deep learning models that can learn representations transferable across subjects (Kindermans et al., 2014). Moreover, few-shot learning has been applied to facilitate the BCI systems’ adaptability with minimal subject-specific data (An et al., 2023). Combining these transfer learning strategies with Riemannian approaches can potentially overcome the generalizability issues posed by inter-subject variability. Nonetheless, for these methods to be truly effective, a larger and more diverse dataset is essential. The current limitations posed by small sample sizes and the heterogeneity of stimuli across experiments necessitate the creation of comprehensive datasets, which employ uniform stimuli to ensure consistency and facilitate more generalized outcomes (Barachant et al., 2013; Rodrigues et al., 2019).

In this study, we develop a comprehensive dataset that can address the challenges associated with inter-subject variability in ERP-based BCIs. The dataset is collected from a large number of subjects under diverse stimulus presentation environments, ranging from liquid crystal display (LCD) displays to AR. Based on this dataset, we introduce two transfer learning approaches. The first approach, termed ‘within-paradigm transfer learning,’ focuses on generalizing the BCI model within the same stimulus presentation paradigm. The second approach, termed ‘cross-paradigm transfer learning,’ seeks to adapt a BCI model built from a specific paradigm to the data in other distinct paradigms. These approaches aim to enhance the adaptability and efficiency of BCIs across varied subjects and environments.

The dataset introduced by this study provides a rich resource for exploring novel transfer learning methods and investigating the nuances of ERP responses across different conditions. By making this dataset publicly available, we aim to stimulate further research of ERP-based BCIs and enable the development of more effective and generalizable BCI systems. This initiative aligns with the emphasis on open science and data sharing, which is crucial for accelerating advancements in multidisciplinary fields such BCI research. In summary, this study aims to contribute significantly to the field of BCI by providing an open dataset that can facilitate the development of algorithms and methodologies for effectively addressing the challenges of inter-subject variability.

## 2 Materials and methods

### 2.1 Subjects

In this study, a total of 84 healthy volunteers participated, each involved in only one experiment, ensuring no overlap of subjects between experiments. The demographic information of subjects is listed in Supplementary Table 1. The distribution of subjects across five different online BCI experiments was as follows: 30 subjects were recruited for the TV control experiment, 15 in the door lock (DL) control, and another 15 in the electric light (EL) control. Additionally, 14 subjects participated in the bluetooth speaker (BS) control experiment, while 10 were involved in the air conditioner (AC) control experiment. This distribution confirms that transfer learning applied within or between paradigms did involve transfer between subjects, given the distinct participant groups for each experiment. Compared to prior research where the subject pool ranged from 5 to 18 individuals, our study maintained a similar scale in terms of the number of subjects for each experiment (Serby et al., 2005; Townsend et al., 2010). Ethical approval for this study was granted by the Institutional Review Board at the Ulsan National Institutes of Science and Technology (IRB: UNISTIRB-18-08-A), and all subjects provided informed consent before participating.

### 2.2 EEG data acquisition

**FIGURE 1:**
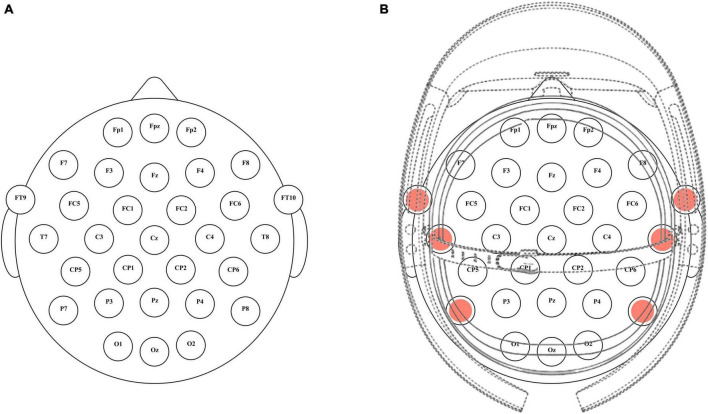
The placement of electrodes and the experimental settings for two different environments. **(A)** Electrode configuration for the LCD environment using 31 electrodes based on the 10–20 system. **(B)** Electrode configuration for the AR environment using 25 active electrodes. The omitted 6 channels (FT9, FT10, T7, T8, P7, P8) are represented by red dots.

Scalp EEG data were collected using a commercially available EEG acquisition system (actiCHamp, Brain Products GmbH, Germany) following the electrode placement guidelines of the American Clinical Neurophysiology Society’s 10–20 system. In the LCD experimental environment, data were acquired from 31 active wet electrodes positioned (FP1, FPz, FP2, F7, F3, Fz, F4, F8, FT9, FC5, FC1, FC2, FC6, FT10, T7, C3, Cz, C4, T8, CP5, CP1, CP2, CP6, P7, P3, Pz, P4, P8, O1, Oz, and O2) at specific locations on the scalp (Figure 1A). For the augmented reality (AR) environment, facilitated by Microsoft’s HoloLens 1, six channels were omitted, resulting in 25 active electrodes at different scalp locations (FP1, FPz, FP2, F7, F3, Fz, F4, F8, FC5, FC1, FC2, FC6, C3, Cz, C4, CP5, CP1, CP2, CP6, P3, Pz, P4, O1, Oz, and O2) (Figure 1B). In both settings, reference and ground electrodes were placed on the linked mastoids of the left and right ears, respectively. The impedance of the electrodes was maintained below 5 kΩ. The EEG signals were digitized at a sampling rate of 500 Hz and band-pass filtered between 0.01 and 50 Hz.

### 2.3 Experimental design

**FIGURE 2:**
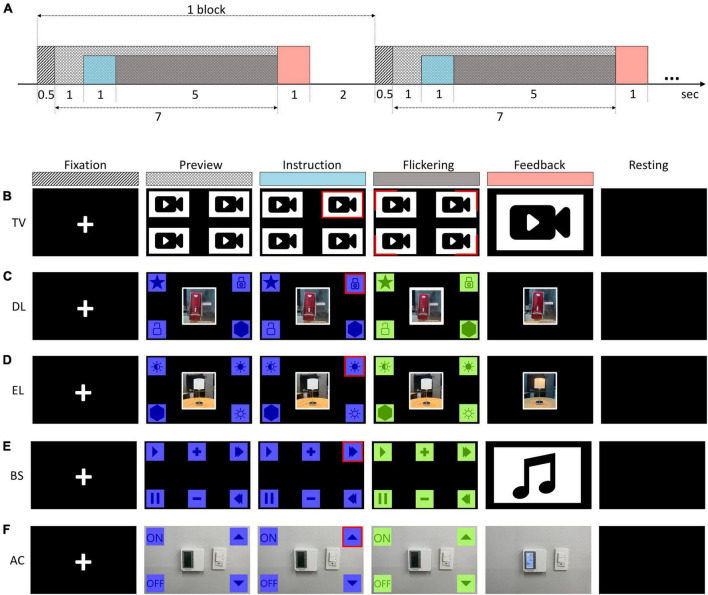
Various BCI paradigms used for controlling different home appliances: **(A)** Temporal sequence of the experiment paradigm, **(B)** TV, **(C)** Door Locks (DL), **(D)** Electric Lights (EL), **(E)** Bluetooth Speakers (BS), and **(F)** Air Conditioners (AC).

All the BCI experiments for home appliance control shared an identical experimental procedure. The experiments consisted of a series of blocks of the oddball task (Figure 2A). A single block began with 0.5-s fixation where a white cross appeared at the center of the screen. The preview of stimuli followed for 1 s which allowed subjects to perceive all the stimuli. Then, a target stimulus was instructed by changing the color of the border of the target stimulus to red for 1 s. Each stimulus flickered in a random order, which took 5 s total. Afterward, the feedback of BCI control was shown for 1 s, and subjects rested for 2 s until the next block. During the training phase, 50 blocks were repeated, while in the testing phase, 30 blocks were repeated. Specific paradigms for each home appliance are described below (see also Figures 2B–F).

To develop a BCI for controlling TV channels, an emulated Multiview TV platform was created. This platform displayed four preview channels simultaneously at four quadrants from the center of the screen (Figure 2B). The video clips in each channel served as both channel previews and visual distractors. The video clips were presented on a 50-inch Ultra High Definition (UHD) TV with a resolution of 1,920 × 1,080 and a refresh rate of 60 Hz. Additional red stimuli flickering at a frequency of 8 Hz surrounded the corners of the video clip windows. Each block began with the subject fixating their gaze at the center of the screen, followed by an instruction indicating the target channel by turning its boundary red. Subsequently, the four video clips and their surrounding stimuli were displayed, each flickering ten times in a random order. Subjects were instructed to gaze at the stimulus surrounding the target channel while seated comfortably 2.5 meters away from the TV screen. The video clip of the selected channel was then displayed for 1 s as feedback.

The BCI experiments for controlling door locks (DL) and electric lights (EL) presented the stimuli on a tablet PC screen (Figures 2C, D). Subjects were instructed to select one of the two control icons for door lock control (lock/unlock) or one of three icons for electric light control (on/off/dim). To maintain a target-to-non-target stimulus ratio of 1:3 (4 classes), dummy stimuli were added to the task. Although the number of commands for TV, EL, and DL varied, dummy stimuli were added to ensure that the classifier could differentiate among four targets (target-to-non-target stimulus ratio of 1:3). Additionally, a shared characteristic among all three experiments was the utilization of an LCD environment for stimulus presentation. Consequently, these experiments were categorized under a unified paradigm referred to as the 4-class LCD paradigm. The BCI experiment to control Bluetooth speakers (BS) was conducted in which subjects selected one of the six functions (on/off/play/pause/next track/previous track). In this experiment, the target-to-non-target stimulus ratio was maintained at 1:5 (Figure 2E). A BCI experiment was conducted to control air conditioners (AC) through an AR environment. The AR stimuli were displayed on Microsoft’s HoloLens 1, which had a resolution 2 HD 16:9 light engines–2.3 million light points. The BCI for AC control consisted of four functions (on/off/ + temperature/- temperature). The target-to-non-target stimulus ratio was maintained at 1:3 (Figure 2F).

The stimuli were presented in the form of icons representing control functions in the four experiments (DL, EL, BS, and AC). All the stimuli were displayed in blue. Then, during the stimulus flickering period, each stimulus flickered in a random order by briefly changing its color to light green for 0.625 s. The inter-stimulus interval was 0.125 s. Each stimulus flickered 10 times in the stimulus flickering period (McFarland et al., 2011). The home appliances to be controlled were displayed in the background of the screen in the DL, El, and AC experiments, while no display of the device was provided in the BS experiment (Figure 2).

### 2.4 Dataset structure

The data acquired from the experiments had a hierarchical structure, comprising three levels. A summary of the dataset information is provided in Table 1. First Level: Home Appliance Type. The first level categorized the data based on the type of home appliance being controlled through the BCI. Second Level: Subject. Within each home appliance category, the data was further divided based on the subject. Each subject was anonymized and identified only by a unique ID. Third Level: Block-Specific Data. The third level contained the granular, block-based data for each subject. The data files were named following a specific convention for easy identification: SubX_training refers to the training data for subject X, and SubX_test_tr_Y refers to the test data for the Y-th block of subject X. Each data file at the third level was composed of two main components:

**TABLE 1:** Summary of dataset information.

Home appliance	Number of subjects	Sex (male/ female)	Age (mean ± std)	Paradigm	Number of functions	Number of Train Blocks	Number of Test Blocks
TV	30	23/7	21.63 ± 2.31	4-class LCD	4	50	30
Door Lock	15	12/3	22.87 ± 2.07	4-class LCD	2	50	30
Electric Light	15	10/5	22.13 ± 2.20	4-class LCD	3	50	30
Bluetooth Speaker	14	9/5	22.64 ± 3.08	6-class LCD	6	50	30
Air Conditioner	10	6/4	22.40 ± 2.59	4-class AR	4	50	30

• Data.signal: it contains EEG signals in a matrix format, with dimensions; [channel x time (points)].
• Data.trigger: it contains the event trigger data in a row vector format, with dimensions; [1 x time (points)].

The trigger types are coded as follows:

• 11: Block start
• 12: Stimulation start
• 13: Block end
• 1 to 6: Types of stimuli
• Between 11 and 12: Indicates the target stimulus.

### 2.5 EEG preprocessing

**FIGURE 3:**
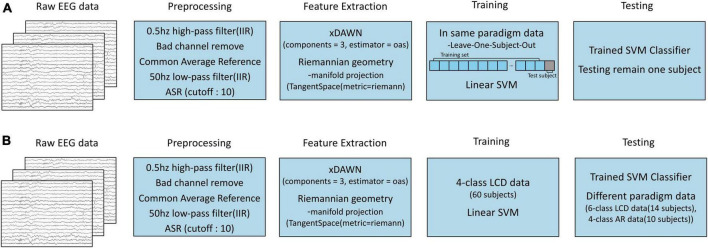
Overall process of transfer learnings. **(A)** Process of within-paradigm transfer learning. **(B)** Process of cross-paradigm transfer learning.

In our analysis, the preprocessing of EEG signals involved several steps. Initially, the EEG signals were subjected to high-pass filtering at 0.5 Hz using a Infinite Impulse Response (IIR) filter to eliminate slow drifts (4th-order Butterworth filter). Subsequently, each channels was evaluated for signal quality; the channels containing EEG signals that had the Pearson correlation coefficient lower than 0.4 after a 2-Hz low-pass filtering (2nd-order Butterworth filter) with more than 30% of all other channels were considered ‘bad’ and subsequently removed (Bigdely-Shamlo et al., 2015). The EEG signals in the remaining channels were then re-referenced using the Common Average Reference (CAR) method (Mullen et al., 2013). Following this, a 50-Hz low-pass IIR filter was employed to reduce line noise (4th-order Butterworth filter). Lastly, Artifact Subspace Reconstruction (ASR) was employed with a cutoff value of 10 for artifact removal (Mullen et al., 2013; Figure 3).

Event-related potentials were extracted by segmenting and averaging EEG signals in epochs time-locked to the stimuli. Specifically, an epoch was defined as 0.2 s before and 0.6 s after the onset of each stimulus.

### 2.6 Online BCI

The online BCI system was trained and tested over separate blocks. The training phase consisted of 50 blocks, during which subjects were instructed to gaze at a randomly displayed target. Feedback was provided based on the desired target stimulus, not the decoded one. From the training data, ERP amplitude features distinguishing target from non-target ERPs were selected using a two-sample *t*-test (*p* < 0.01). Dimensionality reduction was performed using Principal Component Analysis (PCA), retaining components that explained more than 90% of the feature variance. A Support Vector Machine (SVM) classifier with a linear kernel was then constructed to identify the target based on ERP features (Kim et al., 2019).

During the testing phase, which consisted of 30 blocks, subjects controlled a specified home appliance according to the target instruction using the BCI system. The classifier, trained from training phase (50 blocks), used the ERP features to predict the target command, with the prediction outcome then provided as feedback.

### 2.7 Transfer learning

Transfer learning was employed in this study to investigate its feasibility and effectiveness within and across different BCI paradigms (Wang et al., 2015). Among various types of transfer learning, this study focused on transductive transfer learning, in which the target task (classification) is identical between the domains but the training and testing domains differ (Pan and Yang, 2010). When applying transfer learning, we used only testing data that consisted of 30 blocks from all subjects, excluding the training data of 50 blocks from the analysis. The dataset was divided into three distinct subsets based on the experimental paradigms: (1) 4-class LCD (TV, DL, EL), (2) 6-class LCD (BS), and (3) 4-class AR (AC).

Initially, transfer learning was performed within each paradigm. The Riemannian geometry approach was used for feature extraction. We utilized the Riemannian geometry approach for feature extraction, applying the PyRiemann library (Barachant et al., 2023). ERP signals were spatially filtered using xDAWN, configured to use five components (n_components = 5) and an Orthogonal Approximation Subspace (OAS) estimator for covariance estimation (estimator = ‘oas’). Following xDAWN filtering, the signals were projected onto the Riemannian manifold with Tangent Space mapping [TangentSpace(metric = ‘riemann’)], preserving the manifold’s intrinsic structure (Barachant et al., 2013; Figure 3). The projected signals in the Riemannian manifold were used as features for classification. This projection was executed through tangent space mapping, a technique that linearizes the manifold at a given point, transforming the covariance matrices into tangent vectors in a Euclidean space. This projection is not just a transformation; it preserves the intrinsic structure of the manifold in the new Euclidean vector space. A Support Vector Machine (SVM) classifier with a linear kernel was constructed to identify the target based on the extracted features.

The accuracy of the within-paradigm transfer learning classification was evaluated using a ‘Leave-One-Subject-Out’ cross-validation approach for each subject within every dataset. This means that for each cross-validation fold, 30 blocks from all subjects except one were used for training, and the 30 blocks from the excluded subject were used for testing (for instance, in a 4-class LCD subset, training data consisted of 30 × 59 = 1,770 blocks, while test data consisted of 30 blocks from the excluded subject) (Figure 3A).

Next, transfer learning was applied across different paradigms. The feature extraction and classification methods remained consistent with the within-paradigm transfer learning. The larger training data size offered the opportunity to build a more robust model capable of generalizing well to other tasks. Therefore, the largest dataset, which consisted of 60 subjects in the 4-class LCD (TV, DL, EL), served as the training data for cross-paradigm transfer learning. Cross-paradigm transfer learning was then applied to the remaining datasets: 6-class LCD involving 14 subjects (BS) and 4-class AR involving 10 subjects (AC). The accuracy of the classification was evaluated for each subjects (Figure 3B).

## 3 Results

### 3.1 BCI performance

**FIGURE 4:**
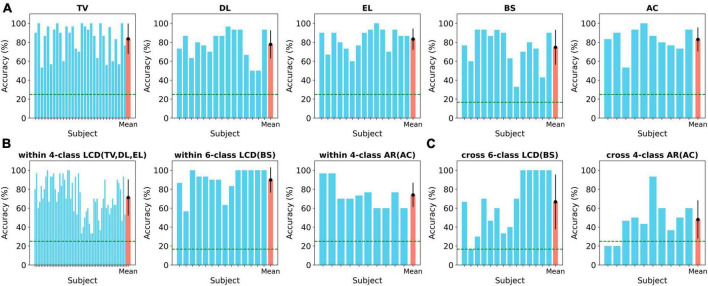
Accuracy across different paradigms and transfer learnings. **(A)** Online classification accuracy for controlling various home appliances in 4-class and 6-class LCD and 4-class AR environments. **(B)** Classification accuracy when applying within-paradigm transfer learning across different appliances. **(C)** Classification accuracy when implementing cross-paradigm transfer learning between 4-class LCD and 6-class LCD, and 4-class AR environments.

The performance of the BCI system was evaluated over three different paradigms, including a 4-class LCD environment (TV, DL, EL), a 6-class LCD environment (BS), and a 4-class AR environment (AC). The primary metric used to assess performance was classification accuracy, which measured the ratio of correctly classified commands to the total number of commands. Figure 4A shows the accuracy of the online control of each appliance. In the 4-class LCD paradigm, the system was used to control three home appliances: TV, DL, and EL. The average classification accuracy for TV control (*N* = 30) was 83.62 ± 16.38% (MEAN ± STD). The accuracy ranged from a minimum of 53% to a maximum of 100%. For DL control (*N* = 15), the average accuracy was 77.78 ± 15.3% (range: 50–96.6%). For EL control (*N* = 15), the average accuracy was 83.35 ± 11.56% (range: 60–100%). Note that the chance level was 25%. In the 6-class LCD paradigm, which focused on controlling a Bluetooth Speaker (BS), the average classification accuracy (*N* = 14) was 74.67 ± 19.2% (range: 33.33–93.33%). In the 4-class AR paradigm, which involved controlling an Air Conditioner (AC), the average classification accuracy (*N* = 10) was 86.3 ± 13.28% (range: 53.3–100%).

There was no significant difference in accuracy among home appliances within the 4-class LCD paradigm (Kruskal–Wallis test, *p* > 0.05). When comparing accuracy between the different paradigms, the 6-class LCD paradigm (BS) and 4-class AR paradigm (AC) showed no significant difference (Kruskal–Wallis test, *p* > 0.05).

### 3.2 Within-paradigm transfer learning

We investigated transfer learning within the same paradigm, as described in the Methods section. Figure 4B shows results of each appliance accuracy when applying within-paradigm transfer learning. In the 4-class LCD environment, the average classification accuracy was 71.17 ± 19.26% (range: 33.33–100%). In the 6-class LCD environment, the average classification accuracy was 89.76 ± 13.87% (range: 56.67–100%). Lastly, in the 4-class AR environment, the average classification accuracy was 74.00 ± 13.59% (range: 60–96.67%). There was no significant difference in accuracy with and without transfer learning in the 4-class AR environment and the 4-class AR environment (Paired *t*-test, *p* > 0.05). But there was a significant increase in accuracy with transfer learning for the 6-class LCD environment (Paired *t*-test, *p* < 0.05). It indicated that we could successfully apply the BCI system built from others’ data to a new user without calibrating the system for the new user.

### 3.3 Cross-paradigm transfer learning

Furthermore, we explored the feasibility of cross-paradigm transfer learning, while maintaining consistency in feature extraction and classification methods used in within-paradigm transfer learning. Figure 4C displays the outcomes of the accuracy of each appliance when implementing cross-paradigm transfer learning. When transferring from the 4-class LCD to the 6-class LCD environment, the average classification accuracy was 66.67 ± 29.99% (range: 16.67–100%). Similarly, when transferring from the 4-class LCD to the 4-class AR environment, the average classification accuracy was 48.00 ± 21.27% (range: 20–93.33%). A significant decrease in accuracy was observed in both cases (Paired *t*-test, *p* < 0.01). Accuracy dropped by 8% from BCI without transfer learning to that with transfer learning for the 6-class LCD paradigm. Also, it dropped by 35% from BCI without transfer learning to that with transfer learning for the 4-class AR paradigm. The accuracy of individual subjects, as well as the mean accuracies and standard deviations across all paradigms, can be found in Supplementary Table 2. We observed increase in classification accuracy when integrating the Riemannian geometric approach with xDAWN for feature extraction compared to using xDAWN alone. Statistically significant improvements were seen in three out of five cases (Supplementary Table 3).

## 4 Discussion

One of the most pressing challenges in the field of BCIs is the issue of inter-subject variability. Our study was explicitly designed to address this problem by constructing a comprehensive dataset that covers various stimulus presentation paradigms for ERP-based BCIs. Our dataset included ERP-based BCI data of 84 subjects with the oddball task used for controlling real-world home appliances online. The various stimulus presentation paradigms would offer opportunities to explore similarities and differences of ERP patterns as well as BCI operations induced by different paradigms. The improved classification accuracy achieved by within-transfer learning, particularly in the 6-class LCD (BS), demonstrates a possibility to mitigate the inter-subject variability. It suggests that BCIs can be generalized across different individuals without a substantial loss in performance.

The idea of transfer learning has become a noteworthy approach in the field of BCIs, especially for tackling the issue of inter-subject variability. Our results by within-paradigm transfer learning are promising with reasonable accuracies for different paradigms. This indicates that once the BCI system is built in one paradigm, the model might be applicable to others similar paradigms with little or no additional training (Lee et al., 2020). This could help reduce the time and resources needed for BCI deployment, potentially making it easier to move from research labs to practical use (Ko et al., 2021).

However, the challenge of inter-subject variability still remains between different paradigms according to our results. We observed that cross-paradigm transfer learning was not as successful as within-paradigm transfer learning. Despite the fact that the oddball task was consistent between the paradigms, it is intriguing to observe decreases in the BCI performance when we applied the BCI system built from LCD-based stimulus presentation to the data with AR-based stimulus presentation, event with the same number of classes. In contrast, there was no change in the BCI performance when we applied the BCI system built using the same AR-based stimulus presentation paradigm. Future research is warranted to identify potential factors that contribute to this variability induced by differences in stimulus presentation environment, such as attention, cognitive load and visual distraction (Souza and Naves, 2021; Gibson et al., 2022). Also, using more advanced machine learning methods, which can handle the complex relationships in the data (Alzahab et al., 2021; Zhang et al., 2021), may be useful to improve cross-paradigm transfer learning.

## 5 Conclusion

We constructed a comprehensive dataset of ERP-based BCIs with a relatively larger number of subjects (*N* = 84). The stimulus paradigms used to elicit ERPs in the oddball task were diverse, with varied number of stimuli (4 and 6) and display types (LCD monitor and AR). The dataset included both the training and testing data to build and assess BCIs. Especially, the testing data contained online BCI control data. each associated with different home appliances. We showcased the utility of the data by applying transfer learning to mitigate inter-subject variability. The results showed that transfer learning of BCIs was successful across subjects within the same stimulus presentation paradigm but limited across the different paradigms. It demonstrated the importance of understanding how different visual stimulations affect ERPs even performing the identical oddball task for the design of ERP-based BCIs. The feasibility of transfer learning, verified in this study when the paradigm of visual stimulus presentation is unchanged, may be useful to reduce subject-dependent calibration for practical use of ERP-based BCIs. We also believe that the dataset of ERP-based BCIs presented in this study can offer opportunities to researchers to develop and test new BCI algorithms to advance ERP-based BCIs to be more accessible and user-friendly.

## Data availability statement

The datasets presented in this study are available in an online repository. The data can be accessed at https://github.com/jml226/Home-Appliance-Control-Dataset.

## Ethics statement

The studies involving humans were approved by the Institutional Review Board at the Ulsan National Institutes of Science and Technology. The studies were conducted in accordance with the local legislation and institutional requirements. The participants provided their written informed consent to participate in this study.

## Author contributions

JL: Data curation, Formal analysis, Methodology, Writing—original draft. MK: Data curation, Methodology, Writing—original draft. DH: Data curation, Methodology, Writing—original draft. JK: Data curation, Methodology, Writing—original draft. M-KK: Data curation, Methodology, Writing—original draft. TL: Data curation, Methodology, Writing—original draft. JP: Data curation, Methodology, Writing—original draft. HK: Data curation, Methodology, Writing—original draft. MH: Data curation, Methodology, Writing—original draft. LK: Funding acquisition, Project administration, Writing—review and editing. S-PK: Funding acquisition, Project administration, Writing—original draft, Writing—review and editing.
