# Supplementary material for: A comprehensive dataset for home appliance control using ERP-based BCIs with the application of inter-subject transfer learning
Source: Front Hum Neurosci. 2024 Feb 1;18:1320457. doi: 10.3389/fnhum.2024.1320457 (PMC10867822; doi:10.3389/fnhum.2024.1320457)
Supplement: Supplementary file 1 [file Table_1.docx]

Supplementary Material

A Comprehensive Dataset for Home Appliance Control using ERP-Based BCIs with the Application of Inter-Subject Transfer Learning

Jongmin Lee^1^, Minju kim^1^, Dojin Heo^1^, Jongsu kim^1^, Min-Ki Kim^2^, Taejun Lee^3^, Jongwoo Park^1^, HyunYoung Kim^4^, Minho Hwang^1^, Laehyun Kim^5*^ and Sung-Phil Kim^1*^

^1^Department of Biomedical Engineering, Ulsan National Institute of Science and Technology, Ulsan, South Korea

^2^The Institute of Healthcare Convergence, College of Medicine, Catholic Kwandong University, Gangneung-si. Republic of Korea

^3^Research and Development Division, Hyundai Motor Company, Uiwang, South Korea

^4^Development Division, Infinityenergy Inc, Seoul, South Korea

^5^Center for Bionics, Korea Institute of Science and Technology, Seoul, South Korea

*** Correspondence:**Laehyun Kim
laehyunk@kist.re.kr
Sung-Phil Kim
[spkim@unist.ac.kr](mailto:spkim@unist.ac.kr)

**Supplementary Tables**

S 1. Demographic information of all subjects

| **Paradigm** | **Home Appliance Type** | **Subject Number** | **Age** | **Sex** |
| --- | --- | --- | --- | --- |
| 4-class LCD | TV | 1 | 22 | M |
|  |  | 2 | 23 | F |
|  |  | 3 | 19 | M |
|  |  | 4 | 24 | M |
|  |  | 5 | 20 | F |
|  |  | 6 | 25 | M |
|  |  | 7 | 25 | M |
|  |  | 8 | 21 | M |
|  |  | 9 | 21 | M |
|  |  | 10 | 19 | M |
|  |  | 11 | 20 | F |
|  |  | 12 | 21 | M |
|  |  | 13 | 21 | F |
|  |  | 14 | 21 | M |
|  |  | 15 | 19 | M |
|  |  | 16 | 18 | M |
|  |  | 17 | 24 | M |
|  |  | 18 | 24 | M |
|  |  | 19 | 20 | F |
|  |  | 20 | 22 | M |
|  |  | 21 | 24 | M |
|  |  | 22 | 18 | F |
|  |  | 23 | 25 | M |
|  |  | 24 | 23 | M |
|  |  | 25 | 22 | F |
|  |  | 26 | 21 | M |
|  |  | 27 | 26 | M |
|  |  | 28 | 18 | M |
|  |  | 29 | 20 | M |
|  |  | 30 | 23 | M |
|  | Door Lock | 31 | 21 | F |
|  |  | 32 | 22 | F |
|  |  | 33 | 20 | M |
|  |  | 34 | 22 | M |
|  |  | 35 | 24 | M |
|  |  | 36 | 27 | M |
|  |  | 37 | 24 | M |
|  |  | 38 | 24 | M |
|  |  | 39 | 24 | M |
|  |  | 40 | 21 | M |
|  |  | 41 | 26 | M |
|  |  | 42 | 20 | F |
|  |  | 43 | 22 | M |
|  |  | 44 | 22 | M |
|  |  | 45 | 24 | M |
|  | Electric Light | 46 | 22 | F |
|  |  | 47 | 26 | F |
|  |  | 48 | 21 | M |
|  |  | 49 | 27 | M |
|  |  | 50 | 23 | M |
|  |  | 51 | 22 | M |
|  |  | 52 | 19 | F |
|  |  | 53 | 19 | M |
|  |  | 54 | 23 | M |
|  |  | 55 | 23 | M |
|  |  | 56 | 22 | F |
|  |  | 57 | 21 | F |
|  |  | 58 | 20 | M |
|  |  | 59 | 22 | M |
|  |  | 60 | 22 | M |
| 6-class LCD | Bluetooth Speaker | 1 | 24 | M |
|  |  | 2 | 21 | F |
|  |  | 3 | 21 | F |
|  |  | 4 | 24 | M |
|  |  | 5 | 19 | F |
|  |  | 6 | 23 | M |
|  |  | 7 | 24 | F |
|  |  | 8 | 20 | M |
|  |  | 9 | 31 | M |
|  |  | 10 | 23 | M |
|  |  | 11 | 25 | M |
|  |  | 12 | 19 | F |
|  |  | 13 | 22 | M |
|  |  | 14 | 21 | M |
| 4-class AR | Air Conditioner | 1 | 25 | F |
|  |  | 2 | 19 | M |
|  |  | 3 | 19 | M |
|  |  | 4 | 24 | M |
|  |  | 5 | 20 | F |
|  |  | 6 | 20 | F |
|  |  | 7 | 24 | M |
|  |  | 8 | 23 | F |
|  |  | 9 | 25 | M |
|  |  | 10 | 25 | M |

S 2. Individual subject accuracy and mean accuracies across all paradigms

| **Paradigm** | **Home Appliance Type** | **Subject Number** | **Accuracy(%)** |
| --- | --- | --- | --- |
| 4-class LCD | TV | 1 | 90.00 |
|  |  | 2 | 100.00 |
|  |  | 3 | 53.30 |
|  |  | 4 | 86.70 |
|  |  | 5 | 96.70 |
|  |  | 6 | 56.70 |
|  |  | 7 | 93.33 |
|  |  | 8 | 100.00 |
|  |  | 9 | 90.00 |
|  |  | 10 | 60.00 |
|  |  | 11 | 96.67 |
|  |  | 12 | 90.00 |
|  |  | 13 | 96.70 |
|  |  | 14 | 73.30 |
|  |  | 15 | 70.00 |
|  |  | 16 | 100.00 |
|  |  | 17 | 96.70 |
|  |  | 18 | 93.20 |
|  |  | 19 | 100.00 |
|  |  | 20 | 86.70 |
|  |  | 21 | 63.30 |
|  |  | 22 | 100.00 |
|  |  | 23 | 86.70 |
|  |  | 24 | 56.00 |
|  |  | 25 | 96.00 |
|  |  | 26 | 60.00 |
|  |  | 27 | 83.34 |
|  |  | 28 | 56.70 |
|  |  | 29 | 100.00 |
|  |  | 30 | 76.60 |
|  |  | **Mean** | **83.62 ± 16.38** |
|  | Door Lock | 31 | 73.30 |
|  |  | 32 | 86.70 |
|  |  | 33 | 63.30 |
|  |  | 34 | 80.00 |
|  |  | 35 | 76.67 |
|  |  | 36 | 70.00 |
|  |  | 37 | 86.70 |
|  |  | 38 | 86.70 |
|  |  | 39 | 96.60 |
|  |  | 40 | 93.30 |
|  |  | 41 | 93.30 |
|  |  | 42 | 66.67 |
|  |  | 43 | 50.00 |
|  |  | 44 | 50.00 |
|  |  | 45 | 93.30 |
|  |  | **Mean** | **77.78 ± 15.30** |
|  | Electric Light | 46 | 90.00 |
|  |  | 47 | 67.00 |
|  |  | 48 | 90.00 |
|  |  | 49 | 80.00 |
|  |  | 50 | 73.30 |
|  |  | 51 | 60.00 |
|  |  | 52 | 76.60 |
|  |  | 53 | 90.00 |
|  |  | 54 | 93.33 |
|  |  | 55 | 100.00 |
|  |  | 56 | 93.33 |
|  |  | 57 | 70.00 |
|  |  | 58 | 93.33 |
|  |  | 59 | 86.70 |
|  |  | 60 | 86.70 |
|  |  | **Mean** | **83.35 ± 11.56** |
| 6-class LCD | Bluetooth Speaker | 1 | 76.67 |
|  |  | 2 | 60.00 |
|  |  | 3 | 93.33 |
|  |  | 4 | 93.33 |
|  |  | 5 | 86.67 |
|  |  | 6 | 93.00 |
|  |  | 7 | 90.00 |
|  |  | 8 | 63.00 |
|  |  | 9 | 33.00 |
|  |  | 10 | 70.00 |
|  |  | 11 | 80.00 |
|  |  | 12 | 73.33 |
|  |  | 13 | 43.00 |
|  |  | 14 | 90.00 |
|  |  | **Mean** | **76.67 ± 19.20** |
| 4-class AR | Air Conditioner | 1 | 83.30 |
|  |  | 2 | 90.00 |
|  |  | 3 | 53.33 |
|  |  | 4 | 93.30 |
|  |  | 5 | 100.00 |
|  |  | 6 | 86.70 |
|  |  | 7 | 80.00 |
|  |  | 8 | 76.70 |
|  |  | 9 | 73.30 |
|  |  | 10 | 93.30 |
|  |  | **Mean** | **76.67 ± 19.20** |
| Within  4-class LCD | TV | 1 | 80.00 |
|  |  | 2 | 96.67 |
|  |  | 3 | 60.00 |
|  |  | 4 | 66.67 |
|  |  | 5 | 83.33 |
|  |  | 6 | 70.00 |
|  |  | 7 | 80.00 |
|  |  | 8 | 93.33 |
|  |  | 9 | 100.00 |
|  |  | 10 | 66.67 |
|  |  | 11 | 93.33 |
|  |  | 12 | 93.33 |
|  |  | 13 | 96.67 |
|  |  | 14 | 80.00 |
|  |  | 15 | 66.67 |
|  |  | 16 | 76.67 |
|  |  | 17 | 96.67 |
|  |  | 18 | 83.33 |
|  |  | 19 | 96.67 |
|  |  | 20 | 90.00 |
|  |  | 21 | 70.00 |
|  |  | 22 | 100.00 |
|  |  | 23 | 100.00 |
|  |  | 24 | 70.00 |
|  |  | 25 | 86.67 |
|  |  | 26 | 56.67 |
|  |  | 27 | 93.33 |
|  |  | 28 | 53.33 |
|  |  | 29 | 96.67 |
|  |  | 30 | 76.67 |
|  | Door Lock | 31 | 33.33 |
|  |  | 32 | 40.00 |
|  |  | 33 | 50.00 |
|  |  | 34 | 56.67 |
|  |  | 35 | 60.00 |
|  |  | 36 | 43.33 |
|  |  | 37 | 33.33 |
|  |  | 38 | 33.33 |
|  |  | 39 | 70.00 |
|  |  | 40 | 66.67 |
|  |  | 41 | 70.00 |
|  |  | 42 | 46.67 |
|  |  | 43 | 36.67 |
|  |  | 44 | 60.00 |
|  |  | 45 | 70.00 |
|  | Electric Light | 46 | 90.00 |
|  |  | 47 | 60.00 |
|  |  | 48 | 63.33 |
|  |  | 49 | 53.33 |
|  |  | 50 | 60.00 |
|  |  | 51 | 70.00 |
|  |  | 52 | 63.33 |
|  |  | 53 | 63.33 |
|  |  | 54 | 90.00 |
|  |  | 55 | 86.67 |
|  |  | 56 | 80.00 |
|  |  | 57 | 46.67 |
|  |  | 58 | 93.33 |
|  |  | 59 | 53.33 |
|  |  | 60 | 53.33 |
|  |  | **Mean** | **71.17 ± 19.26** |
| Within 6-class LCD | Bluetooth Speaker | 1 | 86.67 |
|  |  | 2 | 56.67 |
|  |  | 3 | 100.00 |
|  |  | 4 | 93.33 |
|  |  | 5 | 93.33 |
|  |  | 6 | 90.00 |
|  |  | 7 | 90.00 |
|  |  | 8 | 63.33 |
|  |  | 9 | 83.33 |
|  |  | 10 | 100.00 |
|  |  | 11 | 100.00 |
|  |  | 12 | 100.00 |
|  |  | 13 | 100.00 |
|  |  | 14 | 100.00 |
|  |  | **Mean** | **89.76 ± 13.87** |
| Within 4-class AR | Air Conditioner | 1 | 96.67 |
|  |  | 2 | 96.67 |
|  |  | 3 | 70.00 |
|  |  | 4 | 70.00 |
|  |  | 5 | 73.33 |
|  |  | 6 | 76.67 |
|  |  | 7 | 60.00 |
|  |  | 8 | 60.00 |
|  |  | 9 | 76.67 |
|  |  | 10 | 60.00 |
|  |  | **Mean** | **74.00 ± 13.59** |
| Cross 6-class LCD | Bluetooth Speaker | 1 | 66.67 |
|  |  | 2 | 16.67 |
|  |  | 3 | 30.00 |
|  |  | 4 | 70.00 |
|  |  | 5 | 46.67 |
|  |  | 6 | 60.00 |
|  |  | 7 | 33.33 |
|  |  | 8 | 40.00 |
|  |  | 9 | 70.00 |
|  |  | 10 | 100.00 |
|  |  | 11 | 100.00 |
|  |  | 12 | 100.00 |
|  |  | 13 | 100.00 |
|  |  | 14 | 100.00 |
|  |  | **Mean** | **66.67 ± 29.99** |
| Cross 4-class AR | Air Conditioner | 1 | 20.00 |
|  |  | 2 | 20.00 |
|  |  | 3 | 46.67 |
|  |  | 4 | 50.00 |
|  |  | 5 | 43.33 |
|  |  | 6 | 93.33 |
|  |  | 7 | 60.00 |
|  |  | 8 | 36.67 |
|  |  | 9 | 50.00 |
|  |  | 10 | 60.00 |
|  |  | **Mean** | **48.00 ± 21.27** |

S 3. Comparison of transfer learning results with and without Riemann geometry (RG) method (Mean Accuracy (%))

| **Paradigm** | **xDAWN + RG** | **xDAWN** | **Paired t-test, p-value** |
| --- | --- | --- | --- |
| within 4-class LCD | 71.17 ± 19.26 | 65.00 ± 23.14% | ***** 0.0002** |
| within 6-class LCD | 89.76 ± 13.87 | 88.33 ± 14.49 | 0.4738 |
| within 4-class AR | 74.00 ± 13.59 | 67.67 ± 17.36 | **** 0.0081** |
| cross 6-class LCD | 66.67 ± 29.99 | 57.86 ± 29.69 | *** 0.0195** |
| cross 6-class AR | 48.00 ± 21.27 | 40.67 ± 26.28 | 0.1518 |
